# Homovanillic acid and 5-hydroxyindole acetic acid as biomarkers for dementia with Lewy bodies and coincident Alzheimer’s disease: An autopsy-confirmed study

**DOI:** 10.1371/journal.pone.0171524

**Published:** 2017-02-06

**Authors:** Satoru Morimoto, Masaki Takao, Hiroyuki Hatsuta, Yasushi Nishina, Tadashi Komiya, Renpei Sengoku, Yuta Nakano, Akiko Uchino, Hiroyuki Sumikura, Yuko Saito, Kazutomi Kanemaru, Shigeo Murayama

**Affiliations:** 1 Department of Neuropathology, Tokyo Metropolitan Geriatric Hospital and Institution of Gerontology, Tokyo, Japan; 2 Department of Neurology, Tokyo Metropolitan Geriatric Hospital and Institution of Gerontology, Tokyo, Japan; 3 Department of Neurology and Stroke, Saitama Medical University International Medical Center, Saitama, Japan; 4 Department of Neurology, National Hospital Organization Tokyo National Hospital, Tokyo, Japan; 5 Department of Laboratory Medicine, National Center of Neurology and Psychiatry, Tokyo, Japan; McGill University, CANADA

## Abstract

Dementia with Lewy bodies (DLB) and Alzheimer’s disease (AD) are the two most common causes of dementia. Both pathologies often coexist, and AD patients with concomitant neocortical LB pathology (referred to as the Lewy body variant of AD) generally show faster cognitive decline and accelerated mortality relative to patients with pure AD. Thus, discriminating among patients with DLB, AD, and coincident DLB and AD is important in clinical practice. We examined levels of homovanillic acid (HVA), 5-hydroxyindole acetic acid (5-HIAA), tau, phosphorylated tau (p-tau), and beta-amyloid (Aβ) 1–42 in cerebrospinal fluid (CSF) to evaluate their viability as biomarkers to discriminate among different forms of dementia. We obtained a total of 3498 CSF samples from patients admitted to our hospital during the period from 1996 to 2015. Of these patients, we were able to carry out a brain autopsy in 94 cases. Finally, 78 neuropathologically diagnosed cases (10 AD, six DLB, five DLB with AD, five controls without neurological diseases, and 52 cases with other neurological diseases) were studied. CSF levels of HVA and 5-HIAA were consistently decreased in pathologically advanced Lewy body disorder (LBD; Braak LB stages >3) compared with pathologically incipient LBD (Braak LB stages <2). These results suggest that if an individual has LB pathology in the central nervous system, CSF levels of HVA and 5-HIAA may decrease after the onset of clinical symptoms. In addition, CSF levels of HVA and 5-HIAA decreased with LB pathology, and were especially low in cases of DLB and DLB with AD. Furthermore, the combination of HVA, 5-HIAA, and brain specific proteins t-tau, p-tau, and Aβ 1–42 in CSF were useful for discriminating among DLB, DLB with AD, and AD with high diagnostic accuracy.

## Introduction

Dementia with Lewy bodies (DLB) and Alzheimer’s disease (AD) are the leading causes of dementia in elderly individuals. Approximately 70% of DLB patients have neuropathological changes characteristic of AD [1], and at least 59% of AD patients have Lewy bodies (LB) [2]. Interestingly, AD patients with concomitant neocortical LB pathology (referred to as the Lewy body variant of AD; LBV) generally show faster cognitive decline and accelerated mortality relative to patients with pure AD [3]. Therefore, it is important to discriminate clinically whether individuals have only LB, only AD, or both LB and AD pathology. In addition to showing therapeutic effects for AD, cholinesterase inhibitors have also demonstrated promising effects for treating DLB [4, 5]. Similar to vaccination or antibody therapy for AD [6], advanced treatments for DLB may be developed in the future, such as those targeting α-synuclein [7, 8]. Therefore, accurate diagnosis of DLB is needed to ensure the appropriate medications are provided.

To investigate this issue, we focused on CSF biomarkers, which have been investigated intensively in the case of Parkinson disease (PD), another well-known neurodegenerative condition with devastating consequences for motor control. Previously, many CSF biomarkers, such as total α-synuclein [9–13], phosphorylated α-synuclein, oligomeric α-synuclein [10], DJ-1 [11], neurofilament light chain protein [12], visinin-like protein-1 (VILIP-1) [13], and cocaine and amphetamine regulated transcript (CART) [14] have been reported to be useful for diagnosing preclinical and clinical PD and DLB, and distinguishing DLB from AD.

The main pathophysiological characteristic of PD is the depletion of dopamine (DA) in the nigrostriatal system [15]. Therefore, CSF concentrations of the main metabolites of DA—dihydroxy phenyl-acetic acid (DOPAC) and homovanillic acid (HVA)—are reduced in PD [16, 17]. Interestingly, LB inclusions have been reported not only in the nigrostriatal system but also in the dorsal raphe and locus coeruleus neurons, with a concurrent reduction of both serotonin (5-HT) and noradrenalin levels in the post-mortem brains of PD patients [15, 18, 19]. Tohgi et al. reported a 15%–20% reduction in the CSF levels of 5-HT, tryptophan (precursor of 5-HT), kynurenine, 3-hydroxykynurenine (metabolites of tryptophan), and 5-hydroxyindole acetic acid (5-HIAA, the main metabolite of 5-HT) in PD patients [20–23]. These authors showed that CSF 5-HT levels were negatively correlated with the severity of parkinsonian features (e.g., bradykinesia, rigidity, freezing gait) and decreased with levodopa therapy. They also found that CSF 5-HIAA levels were negatively correlated with akinesia and freezing gait [24]. In individuals with DLB, which is in the same neuropathological disease spectrum with PD, CSF-levels of metabolites are also associated with clinical features.

Several clinical studies have reported that measurements of HVA and 5-HIAA levels in the CSF are useful biomarkers for differential diagnosis of DLB and AD [25–28]. However, because these studies were analyzed solely on the basis of clinical diagnoses, they lacked information on the neuropathological backgrounds of the patients. Verifying the diagnosis neuropathologically is important for confirming the diagnostic utility of HVA and 5-HIAA measurements. The aims of the present study were 1) to compare the levels of HVA and 5-HIAA with the progression of LB pathology, and 2) to clarify the possibility of using HVA and 5-HIAA levels to discriminate among DLB, DLB with AD, and AD. We carried out the analysis by using autopsy-confirmed cases in which CSF samples were obtained before death.

## Materials and methods

### Cases

We obtained a total of 3498 CSF samples from patients that had been admitted to our hospital during the period from 1996 to 2015. Of these patients, we were able to carry out brain autopsies in 94 cases. We excluded subjects with acute and destructive disorders of the central nervous system, such as Creutzfeldt-Jakob disease, hydrocephalus, acute cerebral infarction, intracranial hemorrhage, brain tumor including metastasis, malignant lymphoma, and leukemia. Individuals undergoing levodopa therapy at the time of CSF analysis were also excluded because this treatment affects HVA and 5-HIAA levels [21, 29]. A 31-year-old individual with tauopathy and developmental abnormality was also excluded. Ultimately, we used 78 cases for the present study.

### CSF analysis

CSF was obtained by a lumbar puncture made in the interspace between the third and fourth or fourth and fifth lumbar spinous processes. The lumbar punctures were performed following overnight fasting with the patients laying down for one hour prior to the procedure.

We collected 6–10 ml of CSF from these patients. The first 5 ml was placed into two or three polypropylene tubes (Eppendorf Co., Ltd, Japan), which were immediately frozen and stored at −30°C for no more than 7 days before being stored at −80°C for future analysis.

In the present study, we measured the levels of HVA and 5-HIAA in the stored CSF by using a high-performance liquid chromatography system equipped with 16 electrochemical sensors (CEAS Model 5500, ESA, Bedford, MA, USA). In addition, the levels of tau, phosphorylated tau (p-tau)-181, beta-amyloid (Aβ) 1–42, and internal controls, were measured by enzyme-linked immunosorbent assay (ELISA; Fujirebio Inc. Gent, Belgium) on a routine basis in accordance with the manufacturer’s protocol [25]. The intra-assay coefficients of variation were within 7%.

### Neuropathologic analysis

Brains were examined using our Brain Bank for Aging Research (BBAR) protocol [30]. Briefly, they were fixed in 20% buffered formalin (WAKO, Osaka, Japan) for 7 to 13 days. Sections (6-μm thick) were stained with hematoxylin and eosin, the Klüver-Barrera method, and the Gallyas-Braak method. For immunohistochemistry, we used a Ventana BenchMark XT autoimmunostainer (Roche, Basel, Switzerland) in accordance with the manufacturer’s protocol. For neuropathologic analyses, we used an antibody raised against anti-phosphorylated-tau antibody (AT-8; monoclonal, Innogenetics, Gent, Belgium; 1:100), anti-human Aβ 11–28 antibody (12B2, monoclonal, 1:50 dilution, IBL, Maebashi, Japan), phosphorylated α-synuclein (p-syn) antibody (pSyn#64, monoclonal, 1:20000 dilution, a gift from T. Iwatsubo, Japan), and anti-phosphorylated TDP-43 (p-TDP-43) antibody (pSer409/410, monoclonal, 1:10000 dilution, a gift from M. Hasegawa, Japan). We used Braak neurofibrillary tangle (NFT) staging with the Gallyas-Braak staining method, Braak amyloid staging with immunostaining, and the first consensus guidelines to evaluate neuropathological lesions [31–33].

### Clinico-pathological rating methodology for Lewy body disorder

We defined LBD as cases with LB pathology. To compare HVA and 5-HIAA with the progression of LB pathology, we adopted Braak LB staging and divided LBD cases into LB stages 1 to 2 and 3 to 5 [34].

Additionally, to assess the clinical conditions of LBD, we applied our BBAR LB rating system [30, 35]. This system requires clinical symptoms, gross and microscopic neuropathologic alterations, and LB scores used in the consensus guidelines for the clinical and pathologic diagnosis of DLB [33]. Stages 0 to II in this grading scheme include cases with no LB and asymptomatic LBD cases. Stages III to V correspond to the symptomatic LBD cases of PD, PD with dementia (PDD), and DLB (including DLB with AD). In this staging system, PDD was differentiated from DLB by applying the ‘12-month (1-year)’ rule noted in the consensus guidelines (i.e., if “dementia appears more than one year after the onset of Parkinsonism” PDD is diagnosed) [33].

We compared the CSF levels of tau, p-tau, Aβ 1–42, HVA, and 5-HIAA between symptomatic and asymptomatic LBD cases.

### Neuropathologic classification of AD, DLB, and DLB with AD

Neuropathologic diagnosis of AD was obtained on the basis of the following criteria: 1) Braak NFT stage of IV or above, 2) amyloid deposits of stage C in the Braak staging methodology [31, 32, 36], and 3) BBAR LB stage II or less.

Neuropathologic diagnosis of DLB was defined in accordance with the following criteria: 1) BBAR LB stage of IV or V and 2) Braak NFT stage of III or less [30].

Cases with a neuropathologic diagnosis of DLB with AD fulfilled the following criteria: 1) BBAR LB stage of IV or V, 2) Braak NFT stage of IV or above, and 3) amyloid stage of C in the Braak staging method. The criteria for controls were as follows: no dementia, no LB pathology, Braak NFT stage of 0 or I, Braak amyloid stage of 0 or A, and no other neurodegenerative disorders. Three neuropathologists reviewed the samples from each subject separately, and the final diagnosis was made by consensus.

To assess the possibility of distinguishing cases of DLB and DLB with AD from those of AD, we compared the CSF biomarkers of AD cases to those of DLB, DLB with AD, and control cases.

### Statistical analysis

Statistical analyses were performed by using JMP 11 (SAS Institute Inc., Cary, NC, USA) to perform Fisher’s exact test and the χ^2^ test for comparisons of categorical data, the Kruskal-Wallis test for non-parametric analysis. Following the Shapiro-Wilk normality test, Student’s *t* test and the Tukey-Kramer honestly significant difference (HSD) test were used for parametric analyses. Receiver operating characteristic (ROC) curve analysis and associated statistics were applied to establish diagnostic accuracy and optimal (maximal of the Youden's index) CSF biomarker cut-off levels for discriminating DLB with AD from pure AD. The criterion for statistical significance was set to *p* < 0.05.

## Results

The mean age of death was 79 ± 8.6 years (mean ± SD; range, 42–99). A summary of the demographic characteristics of the patients in our autopsy series is shown in Table 1. In accordance with the neuropathologic results, 78 cases in total were classified by disease state into five controls without neurological disorders, 10 AD, five DLB with AD and six DLB. In addition, there were 52 cases with other neurological disorders such as progressive supranuclear palsy, corticobasal degeneration, motor neuron disease, multiple system atrophy, spinocerebellar ataxia, frontotemporal lobar degeneration, and lacunar infarction. In the present study, we focused on control, AD, DLB with AD, and DLB cases. There were no significant differences in age, duration from the onset of clinical symptoms to obtaining CSF, or duration from obtaining CSF to death between the groups. Additionally, none of the markers were correlated with age (data not shown).

**Table 1:** Characteristics of the diagnostic groups.

Demographic characteristics	Control	DLB	DLB with AD	AD	*p*-value
**Number of cases**	5	6	5	10	
**Age, y**	71.4 ± 16.6	79.2 ± 4.22	87.0 ± 79.75	80.9 ± 10.4	NS (*p* = .234)
**Number of females**	2	1	3	4	NS (*p* = .549)
**Onset to death, years**	NA	7.50 ± 6.22	9.20 ± 4.21	10.0 ± 5.68	NS (*p* = .911)
**Onset to LP, years**	NA	5.17 ± 5.04	5.60 ± 4.39	5.60 ± 5.02	NS (*p* = .528)
**LP to death, years**	2.20 ± 3.83	2.33 ± 2.25	3.60 ± 3.78	4.40 ± 3.24	NS (*p* = .424)

Data are means ± standard deviations. The *p-*values are for differences evaluated by the Kruskal-Wallis test. Gender distribution was analyzed using the χ^2^ test. Abbreviations: DLB, dementia with Lewy bodies; AD, Alzheimer disease; NA, not applicable; LP, lumbar puncture; NS, not significant.

### CSF biomarkers and the progression of LB pathology

**Fig 1:**
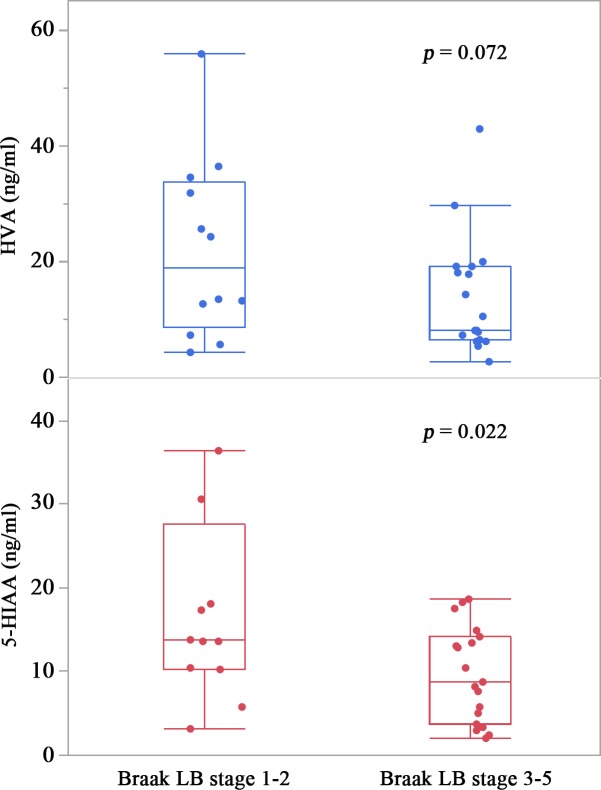
CSF levels of homovanillic acid (HVA) and 5-hydroxyindole acetic acid (5-HIAA). 5-HIAA levels in the pathologically advanced Lewy body disorder (LBD) group (Braak LB stages ≥3) decreased significantly compared with those in the pathologically incipient LBD groups (Braak LB stages ≤2), as assessed with Student’s *t* test. Error bars represent the standard deviations and the bottom, middle, and top lines of the box represent the 25th, 50th (median), and 75th percentiles, respectively. *, *p* < .05.

In pathologically advanced LBD cases (corresponding to Braak LB stages ≥3), CSF levels of HVA [median (interquartile range), 8.1 (6.5–19.1) ng/ml] tended to be lower than in cases of pathologically incipient LBD (corresponding to Braak LB stages ≤2) [19.0 (8.7–33.8) ng/ml] (Student’s *t* test, *p* = .072), and CSF levels of 5-HIAA [8.7 (3.8–14.1) ng/ml] were significantly lower than in cases of pathologically incipient LBD [13.8 (10.3–27.6) ng/ml] (Student’s *t* test, *p* = .022) (Fig 1).

Moreover, CSF levels of tau in advanced LBD cases [135 (94–278) pg/ml] were significantly lower than in cases of pathologically incipient LBD [374 (172–727) pg/ml] (Student’s *t* test, *p* = .004). In contrast, CSF p-tau and Aβ 1–42 levels did not differ significantly between incipient LBD [44 (32–64) and 465 (223–828) ng/ml, respectively] and advanced LBD [47 (33–57) and 457 (309–715) ng/ml, respectively] (Student’s *t* test, *p* = .883 and *p* = .975).

### Discrimination among DLB, DLB with AD, and AD

**Fig 2:**
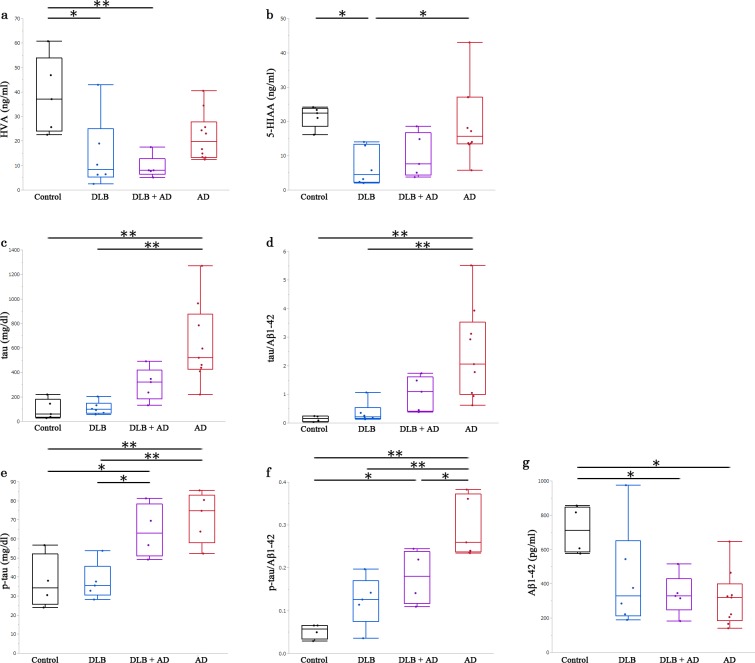
CSF levels of homovanillic acid (HVA), 5-hydroxyindole acetic acid (5-HIAA), tau, phosphorylated tau, and Aβ 1–42. Each panel shows CSF levels of (a) HVA, (b) 5-HIAA, (c) tau, (e) p-tau, and (g) Aβ 1–42 in the cases of control, DLB, DLB with AD, and AD. Panels (d) and (f) are calculated from the values in panels (c), (e), and (g). We analyzed each between-group difference using the Tukey-Kramer honestly significant difference test. Error bars represent the standard deviations and the bottom, middle, and top lines of the box represent the 25th, 50th (median), and 75th percentiles, respectively. *, *p* < .05; **, *p* < .01.

A summary of the CSF data is shown in S1 Table. The average CSF HVA levels in DLB and DLB with AD were significantly lower than in controls (*p* = .013, *p* = .004, respectively) (Fig 2A).

The average CSF 5-HIAA levels in DLB were significantly lower than in controls and AD cases (*p* = .025, *p* = .025, respectively) (Fig 2B). The average CSF tau levels and average tau/Aβ 1–42 ratio in AD were significantly higher than in controls and DLB (tau, *p* = .001 and *p* = .001; tau/Aβ 1–42 ratio, *p* = .009 and *p* = .007) (Fig 2C and 2D). The average CSF p-tau levels in AD and DLB with AD were significantly higher than in controls and DLB cases (AD, *p* = .007 and *p* = .005; DLB with AD, *p* = .045 and *p* = .035) (Fig 2E). The average CSF p-tau/Aβ 1–42 ratio in AD was significantly higher than in controls, DLB, and DLB with AD (controls, *p* < .001; DLB, *p* = .002; DLB with AD, *p* = .041) (Fig 2F). The average levels of CSF Aβ 1–42 in AD and DLB with AD were significantly lower than in controls (*p* = .013, *p* = .043) (Fig 2G). Furthermore, the diagnostic accuracy of CSF HVA and the p-tau/Aβ 1–42 ratio for discriminating AD from DLB with AD were better than that of other markers in this study. An optimal HVA cut-off level of 8.1 pg/mL resulted in area under the ROC curve (AUC), sensitivity, specificity, and diagnostic accuracy levels of .90, .80, 1.00, and .93, respectively. An optimal p-tau/Aβ 1–42 ratio cut-off level of 0.22 resulted in AUC, sensitivity, specificity, and diagnostic accuracy levels of .90, .75, 1.00, and .89, respectively (S1 Fig). When the CSF HVA level is less than 8.1 pg/mL or the p-tau/Aβ 1–42 ratio is less than 0.22, CSF HVA and p-tau/Aβ 1–42 ratio are valuable markers for discriminating DLB with AD from pure AD.

## Discussion

This study has showed that 1) CSF levels of 5-HIAA were significantly reduced, and that of HVA tended to decrease, in cases of pathologically advanced LBD, and 2) the combination of HVA, 5-HIAA, t-tau, p-tau, and Aβ 1–42 in CSF usefully distinguishes among controls, DLB, DLB with AD and AD. Importantly, this study clarified the relationship between ante-mortem lumbar CSF samples and post-mortem brain neuropathologic features.

Dopamine levels in the caudate nucleus and substantia nigra are low in patients with PD [37,38]. Our finding of reduced CSF HVA in LBD is supported by the finding that HVA is also reduced in the brains and CSF of PD patients [26,39–44]. Conversely, 5-HIAA is reported to be normal [44] or reduced in the CSF of PD patients [45]. HVA and 5-HIAA are reduced in the CSF of patients clinically diagnosed with DLB [26,27].In the present study, both HVA and 5-HIAA were low in DLB and DLB with AD. In our results, CSF levels of HVA and 5-HIAA consistently decreased in the pathologically advanced phase of LBD. In general, LBD cases with a Braak LB stage of 3 or above may be symptomatic. These results suggest that if an individual has LB pathology in the central nervous system, CSF levels of HVA and 5-HIAA may decrease after the onset of clinical symptoms of LBD.

Few studies discriminating among cases of DLB, DLB with AD, and AD, have reported CSF levels of biomarkers, including HVA and 5-HIAA, in neuropathologically confirmed cases of DLB complicated with AD [46]. In a study of autopsy-confirmed cases, Weiner and colleagues reported that CSF HVA levels in patients with LBV decreased relative to those in patients with AD, whereas those of 5-HIAA did not [46]. Our results also showed a tendency towards lower HVA levels in DLB with AD than in AD alone (*p* = .06).

However, different pathological diagnostic criteria were used to the current study, as they used CERAD criteria [47] for the diagnosis of AD and Hansen’s criteria [48, 49] for LBV. Our DLB criteria are similar to those of LBV from the viewpoint of LB pathology except for our pathological confirmation of the involvement of peripheral autonomic nervous system by LB pathology in cases of DLB. Moreover, their criteria for AD differed from ours, as they did not estimate NFT, which is important for detailing AD pathology [46]. Although Weiner et al. did not make comparisons with controls, here we showed that CSF HVA levels decreased significantly both in DLB and DLB with AD cases relative to controls.

CSF levels of tau, p-tau, and Aβ 1–42 are now used as clinical biomarkers to diagnose AD [50–53]. However, in clinical practice, discriminating cases of DLB and DLB with AD from those of AD is sometimes difficult. In our cases of AD or DLB with AD, CSF levels of tau, p-tau, and Aβ 1–42 showed relatively typical patterns that have been reported in AD cases previously, although the increase in tau level was mild in cases of DLB with AD. In contrast, CSF levels of tau and p-tau in DLB cases were comparable to those of controls, although those of Aβ 1–42 tended to decrease. CSF levels of tau and p-tau in DLB cases may have been elevated in previous reports [50–53] because AD pathology was not considered in the diagnostic criteria of DLB. In the present study, we strictly excluded AD pathology for diagnosing DLB. Therefore, we were able to estimate the change of each marker in DLB without the effect of AD pathology. Importantly, we revealed that CSF HVA and p-tau/Aβ 1–42 ratio could discriminate DLB with AD from AD with high sensitivity and specificity.

The strengths of the study are to use autopsy confirmed cases and to discriminate the complicated pathologies strictly. A limitation of the study is that the ages of the disease groups are skewed given the small number of cases. Therefore, in future research we will aim to increase the sample size of individuals from whom CSF and pathology are obtained.

## Conclusions

By using autopsy-confirmed cases, we revealed that CSF levels of HVA and 5-HIAA are significant for clinically excluding LB pathology, and that the combination of CSF levels of HVA, 5-HIAA, and brain-specific proteins t-tau, p-tau, and Aβ 1–42 is a strong tool for distinguishing among DLB, DLB with AD, and AD with high diagnostic accuracy. This combination may be a potential biomarker specific for DLB with AD.

## Supporting information

S1 Fig The ROC curves of HVA and p-tau/Aβ1–42 comparing DLB with AD to pure AD. (TIF)

S1 Table The CSF concentrations for each of the biomarkers evaluated. Data are represented by median and interquartile ranges. Abbreviations: DLB, dementia with Lewy bodies; AD, Alzheimer disease; p-tau, phosphorylated tau; Aβ, amyloid β; HVA, homovanillic acid; 5-HIAA, 5-hydroxyindole acetic acid.
(DOCX)
