# Supplementary figures and images for: Homovanillic acid and 5-hydroxyindole acetic acid as biomarkers for dementia with Lewy bodies and coincident Alzheimer’s disease: An autopsy-confirmed study
Source: PLoS One. 2017 Feb 6;12(2):e0171524. doi: 10.1371/journal.pone.0171524 (PMC5293256; doi:10.1371/journal.pone.0171524)

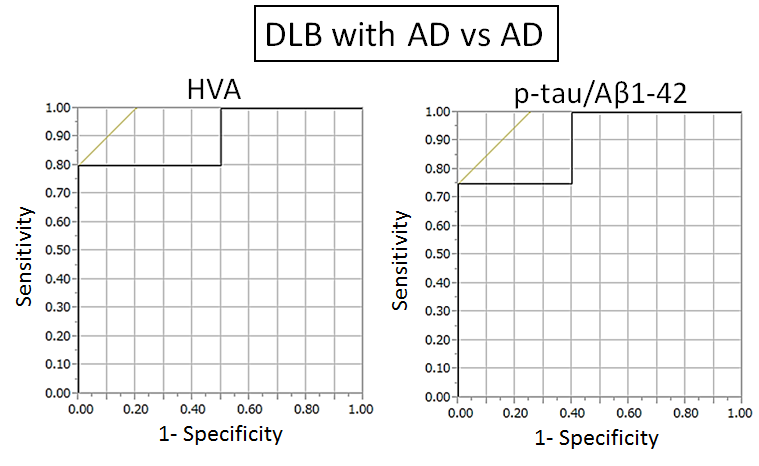

Supplement: S1 Fig — (TIF) [file pone.0171524.s001.tif]
