# Supplementary material for: Homovanillic acid and 5-hydroxyindole acetic acid as biomarkers for dementia with Lewy bodies and coincident Alzheimer’s disease: An autopsy-confirmed study
Source: PLoS One. 2017 Feb 6;12(2):e0171524. doi: 10.1371/journal.pone.0171524 (PMC5293256; doi:10.1371/journal.pone.0171524)
Supplement: S1 Table — Data are represented by median and interquartile ranges. Abbreviations: DLB, dementia with Lewy bodies; AD, Alzheimer disease; p-tau, phosphorylated tau; Aβ, amyloid β; HVA, homovanillic acid; 5-HIAA, 5-hydroxyindole acetic acid. (DOCX) [file pone.0171524.s002.docx]

**Supplementary Table 1. CSF concentrations for each of the biomarkers evaluated.**

|  | n | Control | n | DLB | n | DLB with AD | n | AD |
| --- | --- | --- | --- | --- | --- | --- | --- | --- |
| tau (pg/ml) | 5 | 61 (35–184) | 6 | 101 (67–152) | 5 | 323 (187–421) | 9 | 523 (427–877) |
| p–tau (pg/ml) | 4 | 34 (26–52) | 5 | 36 (31–46) | 4 | 63 (51–78) | 5 | 75 (58–83) |
| Aβ 1–42 (pg/ml) | 4 | 714 (586–847) | 6 | 332 (215–653) | 5 | 332 (250–432) | 9 | 323 (187–400) |
| tau/Aβ1–42 | 4 | .17 (.05–.25) | 6 | .22 (.17–.55) | 5 | 1.10 (.42–1.62) | 9 | 2.07 (1.01–3.54) |
| p–tau/Aβ1–42 | 4 | .06 (.03–.07) | 5 | .13 (.08–.17) | 4 | .18 (.12–.24) | 5 | .26 (.24–.38) |
| HVA (ng/ml) | 5 | 37.2 (24.2–54.0 | 6 | 8.5 (5.4–25.1) | 5 | 8.1 (6.6–12.9) | 10 | 20.0 (13.5–27.9) |
| 5–HIAA (ng/ml) | 5 | 22.5 (18.7–23.9) | 6 | 4.6 (2.3–13.4) | 5 | 7.7 (4.5–16.8) | 10 | 15.7 (13.4–27.1) |

Data are represented by median and interquartile ranges. Abbreviations: DLB, dementia with Lewy bodies; AD, Alzheimer disease; p-tau, phosphorylated tau; Aβ, amyloid β; HVA, homovanillic acid; 5-HIAA, 5-hydroxyindole acetic acid.
